# Natural History of Multiple System Atrophy in North America: A Prospective Cohort Study

**DOI:** 10.1016/S1474-4422(15)00058-7

**Published:** 2015-05-27

**Authors:** Phillip A. Low, Stephen G. Reich, Joseph Jankovic, Clifford W. Shults, Matthew B. Stern, Peter Novak, Caroline M. Tanner, Sid Gilman, Frederick J. Marshall, Frederick Wooten, Brad Racette, Thomas Chelimsky, Wolfgang Singer, David M. Sletten, Paola Sandroni, Jay Mandrekar, the North American Multiple System Atrophy Study Group

**Affiliations:** 1 Department of Neurology, Mayo Clinic, Rochester, MN; 2 Department of Neurology, University of Maryland, School of Medicine, Baltimore, MD; 3 Department of Neurology, Baylor College of Medicine, Houston, TX; 4 Department of Neurosciences, University of California, San Diego, La Jolla, CA (deceased); 5 Parkinson’s Disease and Movement Disorders Center, Pennsylvania Hospital, Philadelphia, PA; 6 Department of Neurology, University of Massachusetts, Worcester, MA; 7 Department of Neurology, University of California, San Francisco, CA; 8 Department of Neurology, University of Michigan, Ann Arbor, MI (retired); 9 Department of Neurology, University of Rochester, Rochester, NY; 10 Department of Neurology, University of Virginia Health System, Charlottesville, VA; 11 Department of Neurology, Washington University School of Medicine, St. Louis, MO and School of Public Health, Faculty of Health Sciences, University of the Witwatersrand, Parktown, South Africa; 12 Department of Neurology, Medical College of Wisconsin, Milwaukee, WI; 13 Division of Biomedical Statistics and Informatics, Mayo Clinic, Rochester, MN

## Abstract

**Background:**

Multiple system atrophy (MSA) is a rare, fatal neurodegenerative disorder exhibiting a combination of parkinsonism and/or cerebellar ataxia with autonomic failure. We report the first North American prospective natural history study of MSA, and the effects of phenotype and autonomic failure on prognosis.

**Methods:**

175 subjects with probable MSA, both MSA-P and MSA-C, were recruited and prospectively followed for 5 years with evaluations every 6 months in 12 centers. Natural history was evaluated by Kaplan-Meier survival analysis. We compared MSA-P with MSA-C and evaluated predictors of outcome. These subjects were evaluated with UMSARS I (a functional score of symptoms and ability to undertake activities of daily living), UMSARS II (neurological motor evaluation), and the Composite Autonomic Symptoms Scale (COMPASS)-select (a measure of autonomic symptoms and autonomic functional status.

**Findings:**

Mean age of symptom onset was 63.4 (SD 8.57) years. Median survival from symptom onset by Kaplan-Meier analysis was 9.8 years (95% CI 8.8-10.7). Subjects with severe symptomatic autonomic failure (symptomatic orthostatic hypotension, urinary incontinence) at diagnosis had a worse prognosis, surviving 8.0 years (95% CI, 6.5-9.5, n=62) while remaining subjects survived a median of 10.3 years (95% CI, 9.3-11.4, n=113). At baseline MSA-P (n=126) and MSA-C (n=49) were not different in symptoms and function, UMSARS I, 25.2 (8.08) vs 24.6 (8.34), p=0.835; UMSARS II, 26.4 (8.77) vs 25.4 (10.51), p=0.7635; COMPASS_select), 43.5 (18.66) vs 42.8 (19.56), p=0.835. Progression, evaluated by change in UMSARS I, UMSARS II, COMPASS_select over the next 5 years, was not significantly different between MSA-P and MSA-C. Median time to death from enrollment baseline was 1.8 (95% CI, 0.9-2.7) years.

**Interpretation:**

Probable MSA represents late-stage disease with short survival. Natural history of MSA-P and MSA-C are similar. Severe symptomatic autonomic failure at diagnosis is associated with worse prognosis.

**Funding:**

National Institutes of Health (P01 NS044233), Mayo CTSA (UL1 TR000135), the Kathy Shih Memorial Foundation, and Mayo funds.

## Introduction

Multiple System Atrophy (MSA) is a neurodegenerative disorder expressing a combination of autonomic failure, parkinsonism and/or cerebellar ataxia,^1^ with a disease annual incidence of 3/100,000 for subjects age 50-99 years.^2^ Disease progression is typically inexorable. The cause of MSA is unknown, although likely linked to alterations in α-synuclein with subsequent formation of glial cytoplasmic inclusion and selective neuronal pathology.^3, 4^ Significant progress has been made to improve certitude of diagnosis. There is excellent agreement between Consensus Criteria^5, 6^ and post-mortem confirmation of diagnosis.^7, 8^ Observational and retrospective studies including autopsy confirmed studies of MSA have provided important information on phenotype and natural history.^1, 9-12^ Validation with prospective studies, however, has been more limited. Earlier studies^13, 14^ did not use validated MSA-specific instruments. Recently, a prospective natural history study of 141 MSA subjects followed over 2 years has provided novel information on MSA natural history in Europe.^15^ We report here a North American prospective study of 175 MSA subjects followed over 5 years. We included both MSA-Parkinsonism (MSA-P) and MSA-Cerebellar (MSA-C) in order to compare their natural history. Key objectives of our study are to determine prospectively 1. the life expectancy of MSA subjects; 2. the influence of phenotype (MSA-P vs MSA-C) on natural history; and 3. prognostic indicators, especially if early onset of autonomic symptoms influenced prognosis.

## Methods

### Subjects and Evaluation

We studied subjects enrolled at twelve U.S. Neurology centers specializing in Movement and/or Autonomic disorders in an observational and risk factor study of MSA.^16^ Subjects were followed biannually. All centers obtained Institutional Review Board approval. All subjects provided written informed consent and met Consensus Criteria for probable MSA.^5, 6^ Each investigator reviewed an UMSARS training video prior to enrolling subjects to ensure scoring consistency across sites. One hundred and seventy five subjects completed a baseline evaluation and were followed every 6 months thereafter for 5 years for available subjects. To minimize problems associated with delayed recall, we provided inclusion/exclusion criteria for both diagnosis and symptoms. Baseline assessments were completed at the study facility and annually onsite thereafter. Questionnaires were sent via mail to subjects at the 6, 18, 30, 42, and 54 month time points; telephone interviews were completed by the enrolling physician to gather UMSARS data if the questionnaire data were not returned.

We followed Consensus criteria^5, 6^ for inclusion and exclusion of MSA and for designation of MSA-P and MSA-C. The full inclusion/exclusion criteria are provided in appendix A. Subjects were classified by MSA subtype based on study examinations, medical records and, as needed, information from the treating physician. Subjects were categorized as MSA-P if they exhibited parkinsonism but no cerebellar features and in whom parkinsonism preceded cerebellar signs by at least one year. For subjects with both cerebellar and parkinsonism, we designated them by onset of first symptom (ataxia or symptoms of parkinsonism). Onset of first symptom was determined from the EMSA-SG minimal data set which details patient symptoms and date of onset to the nearest month when these symptoms first developed. If the dates were not reported by patients, or they had difficulty with recalling onset, we resorted to other sources including relatives, spouses, and medical history to determine the date of onset. MSA-C subjects were defined as those with predominant cerebellar signs but minimal or no parkinsonism in whom cerebellar signs preceded parkinsonism by at least one year. Subjects with severe symptomatic autonomic failure were defined as orthostatic fall in blood pressure (by 30 mm Hg systolic or 15 mm Hg diastolic) or urinary incontinence (accompanied by erectile dysfunction in men) or both. Levodopa responsiveness was defined as a significant and sustained improvement in motor function observed by the patient after drug administration.

#### Baseline evaluation for MSA subjects

All subjects were screened for study enrollment at a baseline evaluation that included the following measures: demographic information, medical history, concurrent medications, neurological examination, mini mental state exam (MMSE), EMSA-SG minimal data set, Unified MSA Rating Scale (UMSARS), Composite Autonomic Symptoms Scale (COMPASS), SF-36 Health Survey, and Consensus Criteria assessment.

#### Follow-up evaluations for MSA subjects

Yearly onsite follow-up examinations and monthly survey data at 6, 18, 30, 42, and 54 months were included and consisted of the following measurements: review of concurrent medications, MMSE, EMSA-SG minimal data set, UMSARS, COMPASS-select, COMPASS-select-change, SF-36 Health Survey, and Consensus Criteria assessment.

#### Instruments

- Unified MSA Rating Scale (UMSARS)^17^ (I. Activities of Daily Living; II. Motor Examination Scale; III. Orthostatic hypotension; IV. Disability Scale). UMSARS I is a functional score of symptoms and ability to undertake activities of daily living consisting of 12 questions.^17^ Each question was scored from 0 to 4, with a higher score indicating a lower functional status. UMSARS II consists of a neurological examination consisting of 14 questions scored from 0 to 4.^17^ UMSARS III is sitting and standing blood pressure. This data was used to define the presence or absence of OH. UMSARS IV is a disability scale with a range of 1 to 4. UMSARS Total is a sum of UMSARS I and UMSARS II.
- Composite Autonomic Symptom Scale (COMPASS).^18^ A full COMPASS of 169 questions in 11 symptom domains was recorded. COMPASS-select is a subset of the full COMPASS consisting of 46 questions in 5 domains (orthostatic intolerance, secretomotor, bladder, constipation, and sleep), leading to a total score between 0 and 125, with a higher score indicating greater impairment.^12^ COMPASS-select has been found to be more appropriate of a measure for assessing MSA patients as such that is what we report in this paper.^12^
- COMPASS-select-change (completed every 6 months while the participant was enrolled in the study) is a derivation of COMPASS-select in which the participants score their change in autonomic symptoms since their last exam.
- Consensus Diagnostic Criteria
- SF-36^19^ is a self-administered questionnaire comprised of 36 questions to evaluate quality of life. There are physical health and mental health components.
- MMSE is a short 30 item screening test administered by an investigator to evaluate cognitive function.
- EMSA-SG (European MSA Study Group)^20^ minimal dataset – This instrument was used in both our study and the EMSA-SG as a means to quantify patient symptoms and duration. See appendix B, provided by courtesy of Dr. Gregor Wenning.

### Statistical analysis

Summary statistics were presented as mean (standard deviation), median (interquartile range) or frequency (percent) where appropriate. Baseline evaluation measures were compared using Mann-Whitney test or Students T-test. The frequencies of symptoms between groups were analyzed using Chi-Square tests when cell counts had ten or more observations. Fisher’s Exact test was used to assess frequencies of symptoms between groups when cell counts were less than ten observations.

Kaplan-Meier analysis curves were used to analyze graphically the interval in years from first symptom onset to death and expressed as median values. Long-rank test statistics were used to determine whether Kaplan-Meier transition curves differed among subgroups. Cox proportional hazards models were used to calculate univariate hazard ratios for shorter survival using age at disease onset as continuous variable and gender, clinical phenotype, and early development of neurologic and autonomic manifestations as categorical variables. Proportional hazards assumption were tested using plots of scaled Schoenfeld residuals against transformed time for each covariate in a model fit using cox.zph function in R version 3.0.2. Statistical significance was defined at P<0.05. False discovery rate corrected p-values were reported as a way to adjust for multiple comparisons in the study. Data analyses were performed using the statistical software SPSS, version 21 and Kaplan Meier curves were generated using R version 3.0.2.

## Role of Funding Source

NIH and Mayo funds supported the development of the study design and its implementation. This included database development, patient recruitment, study visits, and data collection. NIH, Mayo, and Shih Foundation funds supported the study but had no role in data analysis, data interpretation, and drafting of the manuscript. The corresponding author had full access to all the data in the study and had final responsibility for the decision to submit for publication. This decision was done in consultation with the co-authors to whom the dataset is also accessible.

## Results

**Figure 1:**
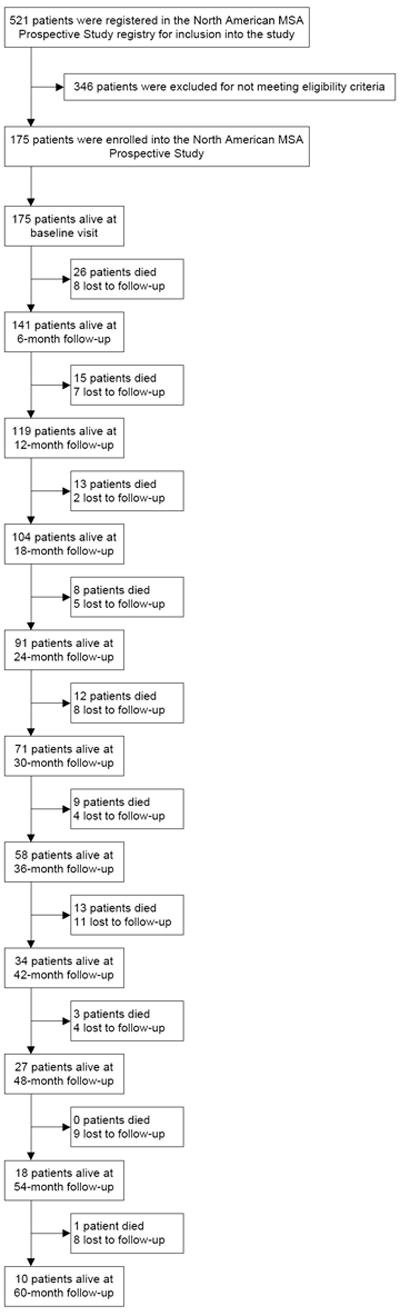
Study flow diagram.

The following sites and principal investigators (number of subjects in brackets) participated in the study: Mayo Clinic, Rochester, Minnesota - Low (30); University of Maryland – Reich (22); Baylor College of Medicine – Jankovic (17); UCSD – Shults (17); University of Pennsylvania – Stern (16); Boston University – Novak (16); Parkinson’s Institute – Tanner (14); University of Michigan – Gilman (14); University of Rochester – Marshall (9); University of Virginia – Wooten (8); Washington University – Racette (8); University Hospital, Cleveland – Chelimsky (4). Enrollment period for the study was December 2003 – May 2008. The last 60-month follow up visit was May 2010. The majority of patients were non-Hispanic, Caucasian men. Education beyond high school was common, with a median of 16 years for both MSA groups. Mean age of MSA at enrollment was 63.4 years. Most patients had MSA-P (72%), with 63% being men (Table 1). Baseline values for symptoms and function (UMSARS I) and deficits (UMSARS II), disability status (UMSARS IV), mental state (MMSE) as well as autonomic symptoms and function (COMPASS-select) were not different between MSA-P and MSA-C (Table 1). Baseline measurements of both components of SF-36 (Physical Health and Mental Health) showed a significant difference between MSA-P vs MSA-C (Table 1). The flow chart (Figure 1) shows the progressive reduction in subjects, mainly due to death beyond 24 months.

Clinical features showed significant differences in a number of domains (Table 2). Autonomic failure was uniformly present in both MSA-P and MSA-C groups. The major autonomic manifestations of orthostatic hypotension, neurogenic bladder (incontinence or incomplete bladder emptying), and constipation were present in >80% of subjects. OH was common in both phenotypes, with MSA-P having OH more commonly than MSA-C (82.5% vs 67.4%, p=0.0541). Medications to treat OH, depression, parkinsonism (in MSA-P), and neurogenic bladder was common (>40%) as was dietary supplements. Only levodopa was significantly different between MSA-P from MSA-C.

As expected, parkinsonian symptoms and cerebellar manifestations were more common in MSA-P and MSA-C, respectively. There is some merging of parkinsonism and especially cerebellar symptoms likely reflecting the late stage of disease. Of note is that 51.6% of patients derived some benefit from levodopa, which lasted a mean duration of 3.2 years.

We evaluated hazard ratios for key clinical features and scores from onset to death (Table 3). The evaluation was on the effect of these variables at baseline on outcome. There was no effect of gender or age. None of the variables had an effect on outcome.

Progression of symptoms and deficits showed a slope of 0.3 (95% CI, 0.2-0.4) from baseline to 12 months and a slope of 0.3 (95% CI, 0.2-0.5) for 12 to 24 months for UMSARS I. UMSASRS II showed a slope of 0.5 (95% CI, 0.4-0.6) from baseline to 12 months and a slope of 0.3 (95% CI, 0.2-0.5) for 12 to 24 months. Beyond 24 months, data were degraded by reduced numbers of cases (mainly due to death) and the curve flattened out. Similarly autonomic symptoms, based on COMPASS-Select-Change, at 41.0 (SD, 31.611) at 6 months changed at a steady rate of about - 0.2 (95% CI, -0.7-0.3) points per month for the next 18 months. Values and change in UMSARS scores from baseline are shown in Table 4.

**Figure 2:**
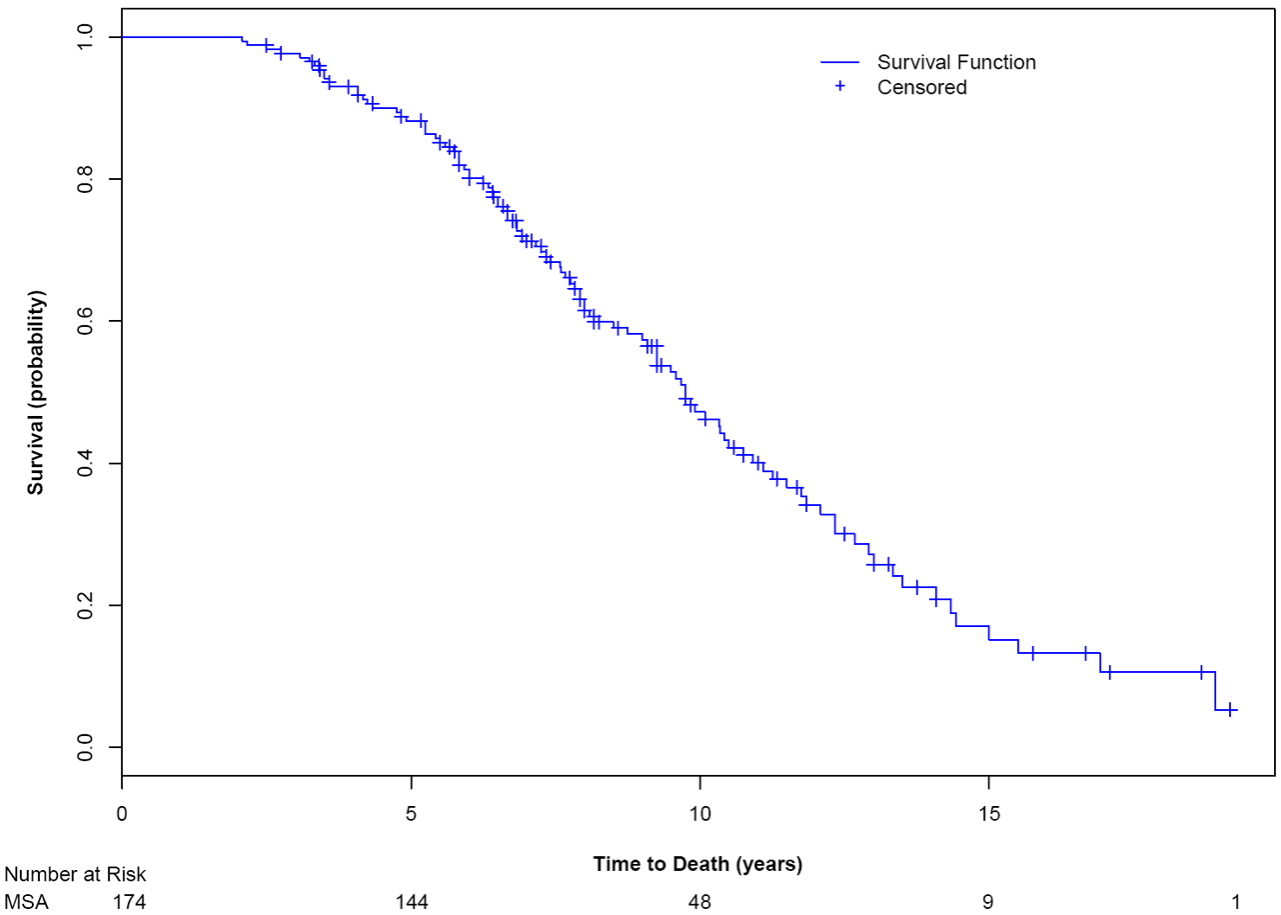

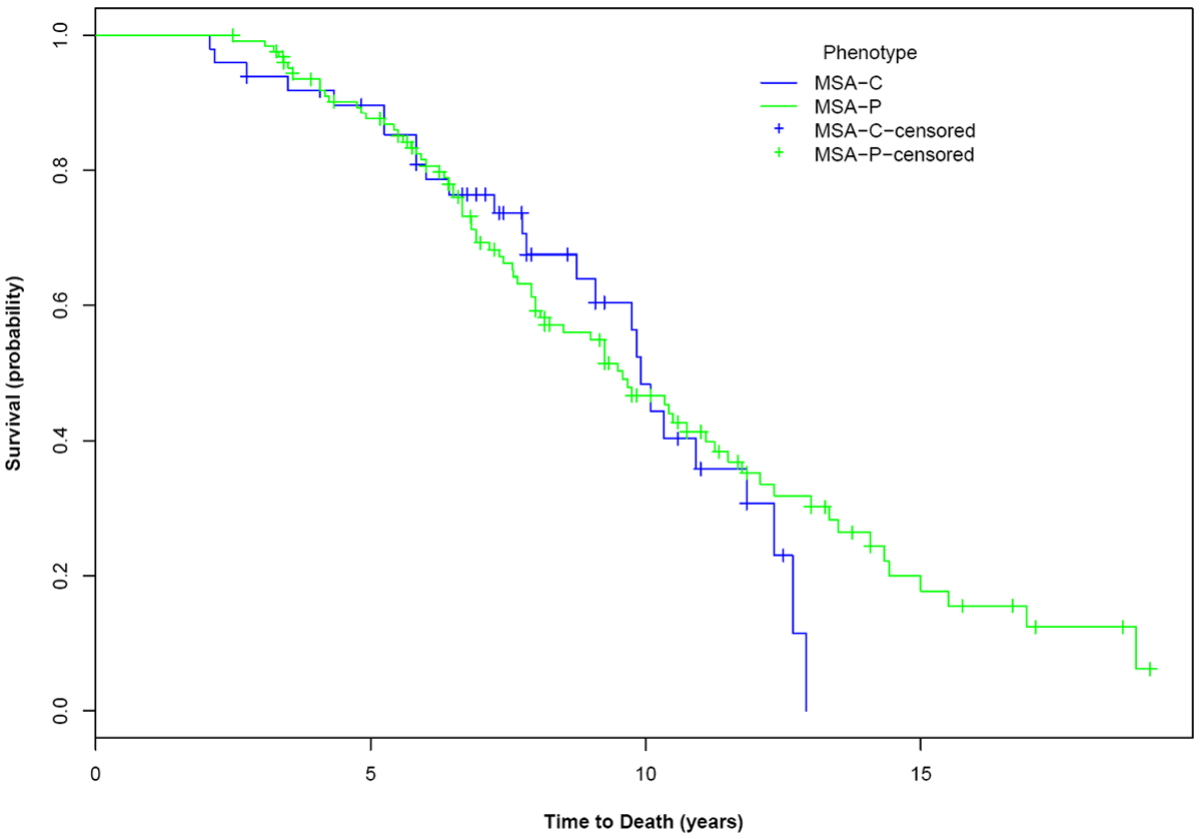

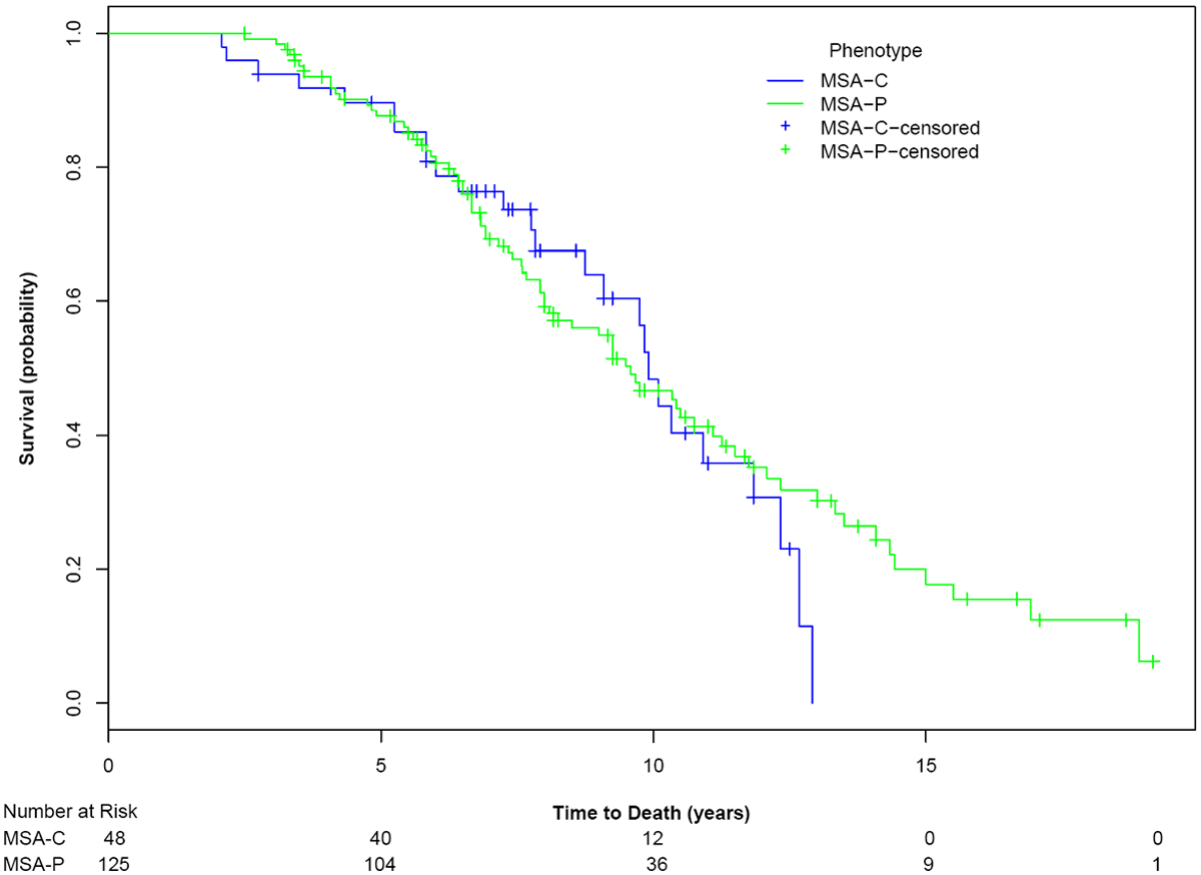

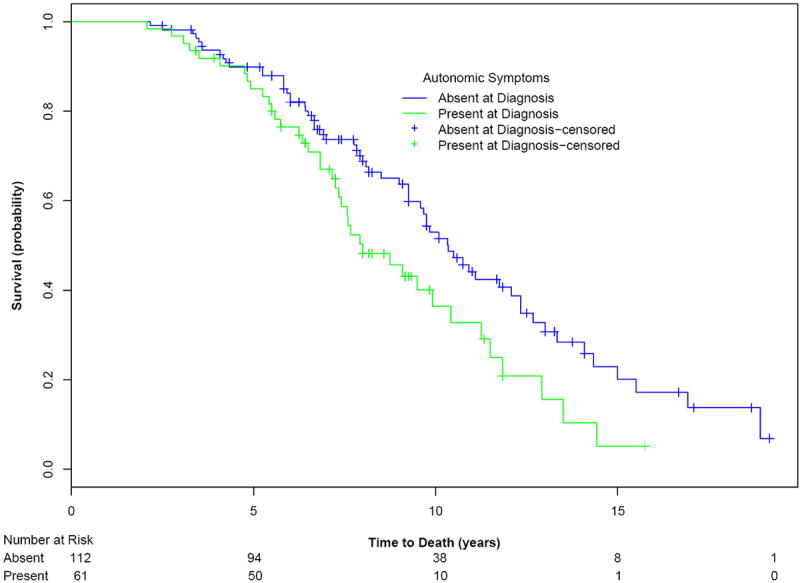
Kaplan-Meier survival curves for probability of dying (upper curve) in all subjects with MSA; and (middle curve) in subjects with MSA-P compared with those with MSA-C, MSAP, and (lower curve) subjects with severe symptomatic autonomic failure at diagnosis compared with those without severe symptomatic autonomic failure at diagnosis. Number at risk of subjects for selected time-points is provided.

During the 5 year study period, we recorded 102 deaths in the study population of 175 subjects. Neuropathological confirmation of the clinical diagnosis was made in all of the 16 subjects who underwent post-mortem verification. On Kaplan-Meier survival analysis (figure 2-upper curve), the median duration of illness from symptom onset to death was 9.8 (95% CI, 8.8-10.7, n=175) years. There were no differences in the median survival time between those with MSA-P and MSA-C (9.6 [95% CI, 8.0-11.2, n=126] vs 9.9 [95% CI, 9.4-10.4, n=49] years; P=0.602; (figure 2-middle curve). Subjects with severe symptomatic autonomic failure at diagnosis, defined as the presence of symptomatic orthostatic hypotension, neurogenic bladder (incontinence or inability to void), or fecal incontinence at initial diagnosis, developing within 12 months of MSA diagnosis, had shorter median survival time (8.0 [95% CI, 6.5-9.5, n=62] years) compared to those without severe symptomatic autonomic failure 10.3 [95% CI, 9.3-11.4, n=113] years; P=0.021 (figure 2-lower curve). We designated the duration of 12 months to exclude subjects with longstanding less-specific autonomic symptoms. Median time to death for all subjects from enrollment was 1.8 (95% CI, 0.9-2.7) years, n=102. Median time to death for MSA-P from enrollment was 1.7 (95% CI, 0.9-2.9) years, n=76. Median time to death for MSA-C from enrollment was 2.0 (95% CI, 1.1-2.5) years, n=26.

## Discussion

The main findings of this prospective study are that MSA-P and MSA-C have a similar natural history with a median duration from onset to death of 9.8 years. Symptoms (UMSARS I) and deficits (UMSARS II) were not different at baseline, and median time to death was only 1.8 years. This suggests that the Consensus Criteria for probable MSA^5, 6^ ensures high diagnostic accuracy but achieves this at a late stage of the disease. The development of severe symptomatic autonomic failure at diagnosis was predictive of a worse prognosis, reducing life-span by 2.3 years.

The North American study, together with the European study,^15^ comprise the only prospective studies on MSA evaluated with disease specific validated instruments. Particular strengths of the two studies are the shared minimal dataset at baseline and the shared instruments to evaluate a range of symptoms and deficits (Table 5). Together they comprise over 300 subjects with this rare disease. The North American study differed from the European study in the duration of study (5 years vs 2 years) and in the certitude of diagnosis (100% vs 77%) (Table 5). A requirement for inclusion was probable MSA in our study whereas the European study accepted both possible and probable MSA. The number of subjects was similar in the two studies (175 vs 141). There was a similar distribution of MSA-P vs MSA-C and gender distribution. Both studies confirmed the dire prognosis of MSA. Remarkably, both studies have found an identical median duration of life from onset to death of 9.8 years (Table 5).

Both prospective studies reported that a large percentage of subjects with MSA-P had a beneficial response to levodopa. Our study reported 56.7% of MSA-P while the European study reported 42.5% benefited. While we recognize that the response maybe suboptimal, it was surprisingly sustained in the 2 studies, with a duration of 3.3 years in our study and 3.5 years in the European study. This observation has clear implications for Consensus criteria of MSA-P and suggests that levodopa responsiveness should not be a requirement in the diagnosis of MSA-P.

There were a number of interesting differences (Table 5). A key finding of the European study was that subjects with the MSA-P had a significantly shorter life-span from baseline to death than those with MSA-C. We did not find a significant difference from symptom-onset or from baseline. One limitation in both our studies is that the number of subjects beyond 2 years is small, due to the high mortality rate (Figure 1). Of note is that the largest retrospective study to date, published in abstract only,^21^ and the autopsy confirmed MSA studies did not find a difference in prognosis by MSA type. It is plausible that the shorter duration of life from baseline relates to delayed diagnosis of MSA-P.^21^ In the retrospective Mayo study by Coon et al,^21^ 685 subjects were evaluated with MSA with follow up, and found that survival from symptom onset to death was identical for MSA-P and MSA-C, but was significantly shorter for MSA-P from baseline. We surmised that the short duration from diagnosis (and baseline) to death for MSA-P relates to the delay in diagnosing MSA-P (retaining diagnosis of parkinsonism) because of the dire outlook with MSA. A second major difference is that a key finding in our study is the worse prognosis of subjects with severe symptomatic autonomic failure at diagnosis (symptoms of orthostatic hypotension, neurogenic bladder, or fecal incontinence) compared with those without severe symptomatic autonomic failure. This is similar to the findings from a recent study of autopsy-confirmed MSA.^8^ Kaplan-Meier curves were significantly different for MSA subjects demonstrating generalized autonomic failure on autonomic testing and also in subjects with neurogenic bladder within 3 years of onset of disease. The European study found a number of variables that suggested a worse prognosis. We did not find an effect of either age or gender or variables that predicted a worse prognosis from baseline to death. It is possible that this apparent discrepancy relates to the more advanced disease related to probable MSA at baseline. One limitation of both studies is the retrospective nature of defining symptom onset. This introduces a recall bias. We have attempted to minimize this bias by predetermining what constitutes symptoms of MSA and what does not, using the predefined minimal dataset. For instance, we did not accept erectile dysfunction, anosmia, constipation or REM sleep behavior as symptom onset. Instead we accepted only symptoms that were more specific for MSA and showed progression over time, such as neurogenic bladder or orthostatic hypotension. We also defined specified symptoms of neurogenic bladder as urinary incontinence or inability to void, discarding more trivial urinary symptoms.

This is the largest prospective study thus far to examine outcome measures in MSA patients. One strength of this study is that the study population consists entirely of patients with a diagnosis of probable MSA. Sixteen of the subjects died and all had their MSA confirmed by an autopsy. Nevertheless, as only patients with probable MSA are included in this study, we expect the potential for misdiagnosis to be low. For instance in an autopsy-confirmed MSA study of 29 subjects with autopsy confirmed MSA, 28/29 had the correct diagnosis of probable MSA antemortem.^7^ The single exception had the phenotype of PAF in the single visit at Mayo Clinic and subsequently evolved into MSA. Another limitation of this research is it is not a population based study, in that patients are recruited tertiary movement disorder or autonomic centers. As such, results might not be generalizable to all USA based MSA patients. One of the limitations of this research is the fall-off beyond year 2, due to the high mortality rate.

Our findings on rate of progression, which are similar to those in the European study and a recently completed Rifampicin study,^22^ have implications for the powering of randomized clinical trials. Considering only patients with a diagnosis of probable MSA for a potential therapeutic trial has disadvantages as well. Patients with probable MSA with a higher UMSARS score than possible MSA^11, 22^ have a flat slope in rate of change (Table 5), accounting for the very large number of subjects needed to power a randomized treatment trial of MSA using probable MSA.^16^ In contrast, selection of subjects who are at an earlier stage of the disease results in a steeper slope and smaller number of subjects needed to power such a study (Table 5).^22^ In the Rifampicin study, we imposed an entry criterion of UMSARS I≤16 (minus question 11), and observed a mean rate of change in UMSARS I score in the placebo group of 0.5 points (SD 0.5) per month. Using these data and assuming an equal SD in the treatment group, 64 participants would be required per group to detect a difference of 50% (ie, a slope of 0.5 points per month in the placebo group *vs* 0.25 points per month in the treatment group) with 80% power and an alpha level of 0.5 based on a two-sample *t* test. Required sample sizes for 40% and 30% reduction in slope would have been 100 participants per group and 176 participants per group, respectively. This is a required number of evaluable patients at the end of the study. Assuming a death or dropout rate of 10% we would need to increase the sample size per group to 111 and 196 participants, respectively The number needed to power such a study, using early and milder disease is much smaller than a study of advanced probable MSA.^16^

## Supplementary Material

1

2

## Figures and Tables

**Table 1:** Patient Demographics and Survey Data

	Overall	MSA-P	MSA-C	P-value*^a^,^b^,^c^
N (%)	175 (100)	126 (72.0)	49 (28.0)	-
Sex				0.4518^a^
Women	70 (40.0)	47 (37.3)	23 (46.9)	-
Men	105 (60.0)	79 (62.7)	26 (53.1)	-
Age	63.38 (8.571)	64.79 (8.901)	59.74 (6.419)	0.0009^C^
*Baseline Values:*				
UMSARS I	25.01 (8.134)	25.18 (8.080)	24.57 (8.339)	0.835^C^
UMSARS II	26.09 (9.271)	26.38 (8.770)	25.35 (10.513)	0.7635^C^
UMSARS IV	3.25 (1.122)	4.0 (2.0 – 4.0)	4.0 (2.0 – 4.0)	0.835^b^
COMPASS_select (5 domains)	43.27 (18.864)	43.45 (18.664)	42.79 (19.558)	0.835^c^
MMSE	29.0 (27.0 – 30.0)	29.0 (27.0 – 30.0)	29.0 (27.0 – 30.0)	0.4518^b^
SF-36				
Physical Health Component	142.71 (67.293)	134.05 (63.678)	164.96 (71.785)	0.021^c^
Mental Health Component	201.53 (85.356)	190.82 (85.001)	229.06 (80.746)	0.021^c^

Values displayed are Mean (Std. Dev), median (IQR), or frequency (percent) as appropriate. UMSARS III is a measurement of sitting and standing blood pressures. This data was used to define the presence or absence of OH; absolute values are not shown.

* Multiple comparison adjusted p-values reported use false discovery rate approach.

a Based on Chi-Square Test (where all cells have 10 or more observations) with level of significance set to 0.05, p-value is asymp. sig. (2-tailed).

b Based on Mann-Whitney test with level of significance set to 0.05. P Value is Asymp. Sig. (2-tailed).

c Based on T-test with level of significance set to 0.05.

**Table 2:** Patient Symptoms Reported at Baseline Assessment

	Overall	MSA-P	MSA-C	p-value*^a^,^b^,^c^
**N (%)**	**175 (100)**	**126 (72.0)**	**49 (28.0)**	**-**
**Autonomic Failure**^i^	**166 (94.9)**	**118 (93.7)**	**48 (98.0)**	**0.5227**^b^
Orthostatic Hypotension^ii^	137 (78.3)	104 (82.5)	33 (67.4)	0.0541^a^
Urinary Incontinence	152 (86.9)	112 (88.9)	40 (81.6)	0.2775^b^
Incomplete Bladder Emptying	146 (83.4)	105 (83.3)	41 (83.7)	1.000^b^
Constipation	153 (87.4)	113 (89.7)	40 (81.6)	0.2693^b^
Fecal Incontinence	50 (28.6)	36 (28.6)	14 (28.6)	1.000^a^
Erectile Dysfunction, Males, n=105	99 (94.3)	75 (94.9)	24 (92.3)	0.6849^b^
Any symptom of autonomic dysfunction	175 (100)	126 (100)	49 (100)	-
**Parkinsonism**iii	**159 (90.9)**	**123 (97.6)**	**36 (73.5)**	**0.0003**^b^
Bradykinesia	160 (91.4)	124 (98.4)	36 (73.5)	0.0003^b^
Rigidity	142 (81.1)	115 (91.3)	27 (55.1)	0.0003^a^
Postural Instability	159 (90.9)	120 (95.2)	39 (79.6)	0.007^b^
Resting Tremor	59 (33.7)	48 (38.1)	11 (22.5)	0.0858^a^
Postural Tremor	98 (56.0)	76 (60.3)	22 (44.9)	0.1011^a^
Unilateral Onset	69 (39.4)	54 (42.9)	15 (30.6)	0.1918^a^
Persistent Asymmetry	62 (35.4)	50 (39.7)	12 (24.5)	0.0972^a^
Any symptom of parkinsonism	165 (94.3)	126 (100)	39 (79.6)	0.0003^b^
**Levodopa Treatment**				**0.0003**^a^
Yes	124 (70.9)	104 (82.5)	20 (40.8)	
No	51 (29.1)	22 (17.5)	29 (59.2)	
**Levodopa Response, n=124**				
Beneficial Response	64 (51.6)	59 (56.7)	5 (25.0)	**0.026**^b^
Response Duration (years)	3.2 (2.32)	3.3 (2.33)	2.6 (2.30)	0.6082^c^
**Cerebellar Dysfunction**^iv^	**100 (57.1)**	**51 (40.5)**	**49 (100)**	**0.0003**^b^
Gait Ataxia	100 (57.1)	51 (40.5)	49 (100)	0.0003^b^
Ataxic Dysarthria	85 (48.6)	40 (31.8)	45 (91.8)	0.0003^b^
Limb Ataxia	93 (53.1)	47 (37.3)	46 (93.9)	0.0003^b^
Sustained Gaze-Evoked Nystagmus	41 (23.4)	21 (16.7)	20 (40.8)	0.0025^a^
Any symptom of cerebellar dysfunction	110 (62.9)	61 (48.4)	49 (100)	0.0003^b^
**Corticospinal Dysfunction**	**93 (53.1)**	**62 (49.2)**	**31 (63.3)**	**0.1385**^a^
Babinski sign	59 (33.7)	40 (31.8)	19 (38.8)	0.459^a^
Hyper-reflexia	70 (40.0)	43 (34.1)	27 (55.1)	0.0237^a^
**Concurrent Medications**				
Blood pressure augmenting medication	75 (42.9)	58 (46.0)	17 (34.7)	0.475^a^
Antihypertensive medication	31 (17.7)	21 (16.7)	10 (20.4)	0.7363^a^
Antidepressant or anti-anxiety medication	85 (48.6)	58 (46.0)	27 (55.1)	0.5583^a^
Carbidopa/Levodopa	86 (49.1)	75 (59.5)	11 (22.5)	0.001^a^
Other parkinsonism medication	52 (29.7)	41 (32.5)	11 (22.5)	0.475^a^
Sleep aid or alerting medication	39 (22.3)	24 (19.1)	15 (30.6)	0.475^a^
Constipation medication	45 (25.7)	35 (27.8)	10 (20.4)	0.5583^a^
Bladder medication	80 (45.7)	56 (44.4)	24 (49.0)	0.7363^a^
Erectile dysfunction medication, N=105	7 (6.7)	5 (4.0)	2 (4.1)	1.000^b^
Supplements	78 (44.6)	57 (45.2)	21 (42.9)	0.8622^a^

Values displayed are Mean (Std. Dev) for continuous variables and Frequency (Percent) for categorical variables.

* Multiple comparison adjusted p-values reported use false discovery rate approach.

a Based on Chi-Square test (where all cells have 10 or more observations) with level of significance set to 0.05, p-value is asymp. sig. (2-tailed).

b Based on Fisher’s Exact test (where any cell has less than 10 observations) with level of significance set to 0.05, p-value is exact sig. (2-tailed).

c Based on Mann-Whitney test with level of significance set to 0.05, p-value is asymp. sig. (2-tailed).

i Criterion for autonomic failure in MSA is defined as Orthostatic fall in blood pressure (by 30 mm Hg systolic or 15 mm Hg diastolic) or urinary incontinence (accompanied by erectile dysfunction in men) or both.

ii Orthostatic Hypotension is defined as a drop in SBP of 20 mmHg or a drop in DBP of 10 mmHg.

iii Criterion for parkinsonism in MSA is defined as bradykinesia plus at least one of the following: rigidity, postural instability, tremor (postural, resting or both).

iv Criterion for cerebellar dysfunction in MSA is defined as gait ataxia plus at least one of the following: ataxic dysarthria, limb ataxia or sustained gaze-evoked nystagmus.

**Table 3:** Univariate Cox Regression Analysis for time to death as outcome

Symptom	N	p-value*	Hazard Ratio	95^th^ Confidence Interval
Age	175	0.8151	1.019	.995 – 1.043
Gender	175	0.8151	0.867	.581 – 1.294
Urinary incontinence	175	0.8151	1.186	.597 – 2.355
Fecal incontinence	175	0.8151	0.779	.508 – 1.194
Incomplete bladder emptying	175	0.858	0.923	.531 – 1.606
Parkinsonism onset	175	0.8151	1.397	.567 – 3.444
Cerebellar (ataxia) onset	175	1.000	1.000	.672 – 1.487
Levodopa responsiveness	124	0.8151	0.708	.442 – 1.132
Pyramidal signs	175	0.8151	0.896	.607 – 1.322
Babinski sign	175	0.858	0.947	.634 – 1.417
Hyperreflexia	175	0.8151	0.835	.557 – 1.251
UMSARS I score ≥ 25	175	0.8151	1.245	.816 – 1.898
UMSARS II score ≥ 25	175	0.8151	1.129	.747 – 1.707

* Multiple comparison adjusted p-values reported use false discovery rate approach.

**Table 4:** Survey questionnaire score decline rates comparing follow-up versus baseline values.

	UMSARS I	UMSARS II	UMSARS Total	MMSE*	SF-36 Physical Health Component	SF-36 Mental Health Component	COMPASS Select/COMPASS Select Change**
**Baseline**							
N	175	175	175	173	175	175	175
Score, mean (SD)	25.01 (8.134)	26.09 (9.271)	51.10 (16.325)	29.0	142.71 (67.292)	201.53 (85.356)	43.27 (18.864)
95% CI	23.80 – 26.22	24.71 – 27.47	48.67 – 53.54	27.0 – 30.0	132.67 – 152.75	188.79 – 214.26	40.45 – 46.08
**6-month follow-up**							
N	110	90	90	85	88	88	108
Score, mean (SD)	26.08 (8.123)	28.01 (7.875)	53.97 (14.242)	29.0	132.85 (68.304)	200.20 (84.370)	41.01 (31.611)
95% CI	24.55 – 27.62	26.36 – 29.66	50.98 – 56-95	27.0 – 29.5	118.38 – 147.33	182.32 – 218.08	34.98 – 47.04
Score difference, mean (SD)	1.98 (4.286)	3.00 (4.264)	4.78 (7.181)	0.0	-19.79 (50.541)	-11.15 (75.273)	-
95% CI	1.17 – 2.79	2.11 – 3.89	3.27 – 6.28	-1.0 – 1.0	-30.50 – -9.08	-27.10 – 4.80	-
Mean percent change	8.95	15.54	10.93	-	-7.36	10.65	-
**12-month follow-up**							
N	96	79	79	74	77	77	92
Score, mean (SD)	26.67 (8.207)	28.72 (7.326)	55.11 (13.503)	29.0	125.77 (65.956)	201.73 (80.463)	38.79 (30.066)
95% CI	25.00 – 28.33	27.08 – 30.36	52.09 – 58.14	27.0 – 30.0	110.80 – 140.74	183.47 – 220.00	32.56 – 45.02
Score difference, mean (SD)	3.91 (5.786)	4.56 (4.974)	7.58 (8.389)	0.0	-22.76 (56.665)	-7.50 (69.229)	-
95% CI	2.73 – 5.08	3.44 – 5.67	5.70 – 9.46	-1.0 – 1.0	-35.62 – -9.90	-23.21 – 8.215	-
Mean percent change	21.04	23.88	18.93	-	-11.01	7.46	-
**18-month follow-up**							
N	79	62	62	60	61	61	76
Score, mean (SD)	28.71 (8.051)	29.24 (7.921)	56.76 (13.732)	29.0	127.27 (65.032)	197.69 (75.910)	39.07 (30.027)
95% CI	26.91 – 30.51	27.23 – 31.25	53.27 – 60.25	27.0 – 30.0	110.62 – 143.93	178.25 – 217.13	32.21 – 45.93
Score difference, mean (SD)	5.62 (5.862)	5.57 (6.687)	10.27 (10.470)	0.0	-33.32 (54.653)	-17.86 (86.367)	-
95% CI	4.31 – 6.93	3.87 – 7.26	7.62 – 12.93	-1.8 – 0.8	-47.32 – -19.32	-39.98 – 4.26	-
Mean percent change	28.96	39.38	28.62	-	-16.37	19.49	-
**24-month follow-up**							
N	69	53	53	49	52	52	67
Score, mean (SD)	29.43 (9.029)	31.21 (8.402)	59.72 (16.146)	28.0	125.00 (69.210)	185.32 (88.910)	38.12 (28.412)
95% CI	27.27 – 31.60	28.89 – 33.52	55.27 – 64.17	26.5 – 29.5	105.73 – 144.27	160.57 -210.07	31.19 – 45.05
Score difference, mean (SD)	6.62 (7.129)	7.25 (7.084)	13.09 (13.447)	0.0	-30.74 (61.827)	-28.26 (86.061)	-
95% CI	4.91 – 8.34	5.29 – 9.20	9.39 – 16.80	-2.0 – 1.0	-47.95 – -13.53	-52.22 – -4.30	-
Mean percent change	34.95	37.10	32.65	-	-14.61	13.15	-
**30-month follow-up**							
N	50	40	40	38	37	37	45
Score, mean (SD)	30.46 (8.608)	32.23 (9.071)	61.35 (16.143)	28.0	117.18 (67.312)	193.78 (80.199)	40.70 (29.367)
95% CI	28.01 – 32.91	29.32 – 35.12	56.19 – 66.51	25.8 – 29.0	94.73 – 139.62	167.04 – 220.51	31.88 – 49.52
Score difference, mean (SD)	7.04 (7.217)	9.63 (9.437)	16.80 (15.655)	-0.5	-40.69 (57.383)	-13.48 (78.356)	-
95% CI	4.99 – 9.09	6.61 – 12.64	11.79 – 21.81	-3.0 – 1.0	-59.82 – -21.56	-39.60 – 12.65	-
Mean percent change	39.74	73.29	50.35	-	-19.16	30.08	-
**36-month follow-up**							
N	52	31	31	26	29	29	48
Score, mean (SD)	30.25 (9.778)	30.97 (8.807)	58.97 (17.398)	28.5	119.95 (50.302)	201.20 (71.747)	42.18 (28.602)
95% CI	27.53 – 32.97	27.74 – 34.20	52.59 – 65.35	25.5 – 30.0	100.82 – 139.08	173.90 – 228.49	33.87 – 50.48
Score difference, mean (SD)	8.33 (7.816)	8.48 (7.206)	14.29 (12.820)	-1.0	-37.95 (50.742)	-9.13 (74.272)	-
95% CI	6.15 – 10.50	5.84 – 11.13	9.59 – 18.99	-3.0 – 1.0	-57.25 – -18.65	-37.38 – 19.12	-
Mean percent change	46.16	49.99	40.29	-	-19.87	5.38	-
**42-month follow-up**							
N	27	19	19	18	19	19	27
Score, mean (SD)	29.56 (8.271)	30.63 (9.476)	58.58 (16.416)	28.5	108.90 (46.141)	195.12 (85.437)	49.67 (26.998)
95% CI	26.28 – 32.83	26.06 – 35.20	50.67 – 66.49	27.0 – 30.0	86.66 – 131.13	153.94 – 236.30	38.99 – 60.35
Score difference, mean (SD)	7.67 (7.432)	9.32 (6.617)	14.95 (10.44)	0.0	-49.97 (41.699)	-21.57 (58.729)	-
95% CI	4.73 – 10.61	6.13 – 12.51	9.91 – 19.98	-1.3 – 0.0	-70.07 – -29.88	-49.88 – 6.74	-
Mean percent change	41.73	48.93	37.02	-	-28.89	-4.82	-
**48-month follow-up**							
N	22	16	16	15	16	16	22
Score, mean (SD)	28.00 (8.384)	28.06 (9.497)	55.56 (16.379)	29.0	119.91 (50.285)	188.48 (76.206)	42.43 (26.861)
95% CI	24.28 – 31.72	23.00 – 33.12	46.84 – 64.29	27.0 – 30.0	93.11 – 146.70	147.87 – 229.09	30.52 – 54.34
Score difference, mean (SD)	6.86 (5.330)	7.31 (8.155)	12.81 (9.731)	0.0	-49.03 (62.141)	-24.06 (66.674)	-
95% CI	4.50 – 9.23	2.97 – 11.66	7.63 – 18.00	-1.0 – 1.0	-82.14 – -15.92	-59.59 – 11.47	-
Mean percent change	40.96	46.05	33.51	-	-19.99	-8.42	-
**54-month follow-up**							
N	15	7	7	6	7	7	15
Score, mean (SD)	32.73 (11.373)	27.57 (14.258)	56.00 (26.249)	28.5	113.00 (53.947)	192.24 (97.524)	42.57 (32.874)
95% CI	26.43 – 39.03	14.39 – 40.76	31.72 – 80.28	23.3 – 29.3	63.12 – 162.89	102.04 – 282.43	24.36 – 60.77
Score difference, mean (SD)	7.73 (7.146)	6.14 (8.934)	13.29 (13.022)	-0.5	-38.57 (35.447)	-30.41 (76.792)	-
95% CI	3.78 – 11.69	-2.12 – 14.41	1.24 – 25.33	-5.5 – 1.0	-71.35 – -5.79	-101.43 - 40.62	-
Mean percent change	32.62	23.18	27.03	-	-20.97	-3.24	-
**60-month follow-up**							
N	8	3	3	3	3	3	8
Score, mean (SD)	35.38 (14.212)	40.67 (18.148)	75.33 (34.196)	29.0	103.67 (50.696)	138.28 (67.368)	56.19 (24.394)
95% CI	23.49 – 47.26	-4.41 – 85.75	-9.61 – 160.28	-	-22.27 – 229.60	-29.07 – 305.63	35.79 – 76.58
Score difference, mean (SD)	10.50 (8.799)	16.67 (9.713)	25.00 (18.028)	1.0	-54.17 (57.520)	-97.33 (61.791)	-
95% CI	3.14 – 17.86	-7.46 – 40.79	-19.78 – 69.78	-	-197.06 – 88.72	-250.83 – 56.16	-
Mean percent change	38.49	64.86	45.72	-	-27.76	-41.68	-

* Median and IQR replace Mean (SD) and 95% CI due to non-normal distribution of the data for MMSE.

** Baseline value is reported using COMPASS Select instrument and subsequent time points show summary using COMPASS Select Change instrument.

**Table 5:** Comparison of North American with European Prospective Study

Variable	North American Study	European Study

Study Design	Prospective study of 175 subjects seen every 6 months for 5 years by 12 North American centers	Prospective study of 141 subjects evaluated every 6 months for 2 years by 15 European centers

Subjects	Probable MSA-P and MSA-C	Possible and probable MSA-P and MSA-C

Study Dates	Enrollment: Dec 2003 – May 2008. Last 60 month follow up May 2010	Jan 2003 to July 2004

Evaluated Variables	Defined minimal dataset and disease specific instruments (includes UMSARS I, II; COMPASS)	Same variables

Kaplan-Meier survival	Median 9.8 years; MSA-P=MSA-C from symptom onset to death	Median 9.8 years; MSA-P had shorter survival from baseline to death

K-M Predictors	Severe symptomatic autonomic failure at diagnosis associated with worse prognosis (by 2.4 years)	MSA-P has shorter survival than MSA-C from baseline to death

Rate of Progression	UMSARS I:	UMSARS I: yr 1, 6.5 (0.5/month); yr 2, 2.9 (0.2/month);
	Baseline to 12 months, 0.3/month	UMSARS II: yr 1, 8.2 (0.7/month); yr 2, 5.0 (0.4/month)
	12 to 24 months, 0.3/month	
	UMSARS II:	
	Baseline to 12 months, 0.5/month	
	12 to 24 months, 0.3/month	

Clinical Trial implications	Probable MSA represents late stage (plateau stage) with modest rate of change	Possible and early MSA is associated with greater rate of change

Autopsy Confirmation	16/16 (100%)	2/2 (100%)

Funding Source	NINDS (NS4 4233)	European Union; Oesterreichische Nationalbank and Austrian Science Fund

## *Panel:* Research in context

### Evidence before this study

We searched PubMed with the following search terms:

[(MSA OR “multiple system atrophy”) AND (progression OR survival)] for reports published before April 9, 2015. We found only a single report of a prospective study on MSA (Wenning et al 2013).^15^ The natural history of MSA has been poorly understood. There is a single prospective natural history study of MSA.^15^ Prior to that the only randomized prospective studies used a non-specific Parkinson plus scale that was suboptimal for MSA.^13, 14^ The study by Wenning et al,^15^ published in 2013, was the first natural history study that analyzed survival and prognostic predictors in a large homogeneous cohort of European patients with MSA and using validated disease-specific rating scales.

### Added Value of this study

This 5 year prospective natural history study of subjects with probable MSA is the largest cohort study done over the longest duration. It found a median survival from symptom onset by Kaplan-Meier analysis was 9.8 years (95% CI 8.8-10.7). Subjects with severe symptomatic autonomic failure at diagnosis had a worse prognosis, surviving 8.0 years (95% CI, 6.5-9.5) while remaining subjects survived a median of 10.3 years (95% CI, 9.3-11.4). Natural history of MSA-P and MSA-C are similar. The study has implications for MSA diagnosis and design of clinical trials. Levodopa response is substantive, occurring in 56.7% of MSA-P and lasting 3.3 (2.33) years. Hence the requirement for lack of levodopa responsiveness for diagnosis of MSA is no longer tenable. The rate of progression has reached a plateau with minimal rate of increase in UMSARS I and II so that the study design using probable MSA requires an unacceptably large number of subjects.

### Implications of all available evidence

Our study synergizes with the European study in a number of important ways. The studies were both large cohorts that shared identical disease-specific, validated instruments to evaluate progression of MSA and used identical minimal baseline datasets. The studies are complementary in that the North American study chose probable rather than possible MSA and extended our period of study to 5 years (from 2 years). Our study, although not population-based, selected subjects from all over the United States. Our finding of disease progression is stable whereas the European study reported a slowing of progression in year 2. The difference likely relates to the slow rate of progression related to advanced disease (probable MSA). The value of 0.30 is significantly lower than the slope of 0.50 we found in the Rifampicin study^22^ and has implications for subject selection in future clinical trials. A key finding in our study is that severe symptomatic autonomic failure at diagnosis is predictive of poor survival. The North American study found identical prognosis (from symptom onset) for MSA-P and MSA-C. The observation by the European prospective study for worse prognosis for MSA-P from baseline could relate to delayed diagnosis of MSA-P.^21^
