# Supplementary material for: Natural History of Multiple System Atrophy in North America: A Prospective Cohort Study
Source: Lancet Neurol. Author manuscript; Available in PMC 2016 Jul 1. (PMC4472464; doi:10.1016/S1474-4422(15)00058-7)
Supplement: 1 [file NIHMS698119-supplement-1.pdf]

HUMAN SUBJECTS:

Subjects will be enrolled in the study only if they meet the criteria of clinically probable MSA according to the Consensus Criteria (Gilman et al., 1999).

Inclusion Criteria:
Participants must meet all inclusion criteria in order to be eligible for the study:

1. Participants aged 30-80 years old with a diagnosis of Possible or Probable MSA of the parkinsonian subtype (MSA-P) or cerebellar subtype (MSA-C) according to The Gilman Criteria (2008).
2. Participants who are less than 3 years from the time of documented MSA diagnosis.
3. Participants with an anticipated survival of at least 3 years in the opinion of the investigator.
4. Participants who are willing and able to give informed consent.
5. “Normal” cognition as assessed by MMSE. We will require a value >24.
6. Diagnosis of MSA <3 years.
7. Duration of disease <5 years.
8. UMSARS I score ≤20 omiting question 11).

Exclusion Criteria:
Any of the following conditions will exclude the participant from entering the study:

1. Pregnant or lactating females.
2. UMSARS score >20 on modified UMSARS I (question 11 eliminated).
3. Dementia (DSM-IV criteria - Amer. Psych. Assoc., 1994). The score on the Mini-Mental State Examination must be >24.
4. Subjects who meet any of the following criteria which tend to suggest advanced disease:
   a. Speech impairment as assessed by a score of ≥ 3 on UMSARS question 1.
   b. Swallowing impairment as assessed by a score of ≥ 3 on UMSARS question 2.
   c. Impairment in ambulation as assessed by a score of ≥ 3 on UMSARS question 7.
   d. Falling more frequently than once per week as assessed by a score of ≥ 3 on UMSARS question.

Multiple System Atrophy

Clinical domains, features and criteria used in the diagnosis of MSA. A feature (A) is a characteristic of the disease and a criterion (B) is a defining feature or composite of features required for diagnosis

I. Autonomic and urinary dysfunction
   A. Autonomic and urinary features
      1. Orthostatic hypotension (by 20 mm Hg systolic or 10 mm Hg diastolic)
      2. Urinary incontinence or incomplete bladder emptying
   B. Criterion for autonomic failure or urinary dysfunction in MSA
      Orthostatic fall in blood pressure (by 30 mm Hg systolic or 15 mm Hg diastolic) or urinary incontinence (persistent, involuntary partial or total bladder emptying, accompanied by erectile dysfunction in men) or both

II. Parkinsonism
   A. Parkinsonian features
      1. Bradykinesia (slowness of voluntary movement with progressive reduction in speed and amplitude during repetitive actions)
      2. Rigidity
      3. Postural instability (not caused by primary visual, vestibular, cerebellar, or proprioceptive dysfunction)
      4. Tremor (postural, resting or both)
   B. Criterion for parkinsonism in MSA
      Bradykinesia plus at least one of items 2 to 4

III. Cerebellar dysfunction
   A. Cerebellar features
      1. Gait ataxia (wide based stance with steps of irregular length and direction)

2. Ataxic dysarthria
3. Limb ataxia
4. Sustained gaze-evoked nystagmus

B. Criterion for cerebellar dysfunction in MSA
Gait ataxia plus at least one of items 2 to 4

IV. Corticospinal tract dysfunction

A. Corticospinal tract features
1. Extensor plantar responses with hyperreflexia

B. Corticospinal tract dysfunction in MSA: no corticospinal tract features are used in defining the diagnosis of MSA

Diagnostic categories of MSA. The features and criteria for each clinical domain are shown in Table 1

I. Possible MSA: one criterion plus two features from separate other domains. When the criterion is parkinsonism, a poor levodopa response qualifies as one feature (hence only one additional feature is required).

II. Probable MSA: criterion for autonomic failure / urinary dysfunction plus poorly levodopa responsive parkinsonism or cerebellar dysfunction.

III. Definite MSA: pathologically confirmed by the presence of a high density of glial cytoplasmic inclusions in association with a combination of degenerative changes in the nigrostriatal and olivopontocerebellar pathways.

Exclusion criteria for the diagnosis of MSA

I. History
Symptomatic onset under 30 years of age
Family history of a similar disorder
Systemic diseases or other identifiable causes for features listed in Table 1
Hallucinations unrelated to medication

II. Physical examination
DSM criteria for dementia
Prominent slowing of vertical saccades or vertical supranuclear gaze palsy
Evidence of focal cortical dysfunction such as aphasia, alien limb syndrome, and parietal dysfunction

III. Laboratory investigation
Metabolic, molecular genetic and imaging evidence of an alternative cause of features.
