# Supplementary material for: Natural History of Multiple System Atrophy in North America: A Prospective Cohort Study
Source: Lancet Neurol. Author manuscript; Available in PMC 2016 Jul 1. (PMC4472464; doi:10.1016/S1474-4422(15)00058-7)
Supplement: 2 [file NIHMS698119-supplement-2.pdf]


Examination Date

☐☐ ☐☐ ☐☐☐☐
Month Day Year

Examiner Initials: ____________

Clinical Diagnosis

1. Clinical Diagnosis According to Quinn Criteria
☐ Possible
☐ Probable
☐ Definite

2. Clinical Diagnosis According to Gilman Criteria
☐ Possible
☐ Probable
☐ Definite

Clinical Features

Autonomic Dysfunction

4a. Onset of Autonomic Symptoms (Month)
Enter UNK for Unknown or NA for Not Applicable.
If NA, skip to item 5.
☐ Month

4b. Onset of Autonomic Symptoms (Year)
☐ Year

5a. Blood Pressure Lying (Systolic)
☐ mmHg

5b. Blood Pressure Lying (Diastolic)
☐ mmHg

6a. Blood Pressure after 2 minutes Standing (Systolic)
☐ mmHg

6b. Blood Pressure after 2 minutes Standing (Diastolic)
☐ mmHg

Symptoms (check either ‘Yes’ as present or ‘No’ as absent)

7. Postural Syncope
☐ No
☐ Yes

8. Urinary Incontinence
☐ No
☐ Yes

9. Fecal Incontinence
☐ No
☐ Yes

10. Incomplete Bladder Emptying
☐ No
☐ Yes

11. Male Erectile Dysfunction
☐ No
☐ Yes
☐ Not applicable (i.e. female participant)

Parkinsonianism

12a. Onset of Parkinsonism (Month)
Enter UNK for Unknown or NA for Not Applicable.
If NA, skip to item 20.
[ ] Month

12b. Onset of Parkinsonism (Year)
[ ] Year

Signs and Symptoms (check either 'Yes' as present or 'No' as absent)

13. Bradykinesia
☐ No
☐ Yes

14. Rigidity
☐ No
☐ Yes

15. Postural Instability
☐ No
☐ Yes

16. Resting Tremor
☐ No
☐ Yes

17. Postural Tremor
☐ No
☐ Yes

18. Unilateral Onset
☐ No
☐ Yes

19. Persistent Asymmetry
☐ No
☐ Yes

L-Dopa Response

20. Beneficial L-Dopa response:
☐ No
☐ Yes
☐ Initial, but not continuing response
☐ Not Received

If Yes or Initial beneficial L-Dopa response:

21. Years of Benefit

[ ] Years

If No or Initial beneficial L-Dopa response:

22. Maximum L-Dopa Dose

[ ] mg/day

23a. Onset of Motor Complications (Month)
Enter UNK for Unknown or NA for Not Applicable.
If NA, skip to item 29.
[ ] Month

23b. Onset of Motor Complications (Year)

[ ] Year

L-Dopa Symptoms

24. Wearing Off
☐ No
☐ Yes

25. On/Off
☐ No
☐ Yes

26. Peak Dose Dyskinesias
☐ No
☐ Yes

27. Biphasic Dyskinesias
☐ No
☐ Yes

28. Off-Dystonia
☐ No
☐ Yes

Cerebellar Dysfunction

29a. Onset of Ataxi (Month)
Enter UNK for Unknown or NA for Not Applicable.
If NA, skip to item 34.
[ ] Month

29b. Onset of Ataxia (Year)

[ ] Year

Signs and Symptoms (check either 'Yes' as present or 'No' as absent)

30. Gait Ataxia
☐ No
☐ Yes

31. Ataxic Dysarthria
☐ No
☐ Yes

32. Limb Ataxia
☐ No
☐ Yes

33. Sustained Gaze-Evoked Nystagmus
☐ No
☐ Yes

Corticospinal Dysfunction

Signs and Symptoms (check either 'Yes' as present or 'No' as absent)

34. Extensor Plantar Responses
☐ No
☐ Yes

35. Hyperflexia
☐ No
☐ Yes

If Yes, date of Onset (Month)
Enter UNK for Unknown.
[ ] Month

Date of Onset (Year)
[ ] Year

Overlap Dysfunction

Signs and Symptoms (check either 'Yes' as present or 'No' as absent)

36. Dysphagia
☐ No
☐ Yes

37. Dysarthria
☐ No
☐ Yes

If Yes, date of Onset (Month)
Enter UNK for Unknown.
[ ] Month

Date of Onset (Year)
[ ] Year

38. Unsteadiness with Falls
☐ No
☐ Yes

If Yes, date of Onset (Month)
Enter UNK for Unknown.
[ ] Month

Date of Onset (Year)
[ ] Year

Cognitive and Physiatric Features

Features (check either 'Yes' as present or 'No' as absent)

39. Memory Impairment
☐ No
☐ Yes

40. Apraxia
☐ No
☐ Yes

41. Frontal Lobe Release Signs
☐ No
☐ Yes

42. Utilization/Imitation Behavior
☐ No
☐ Yes

43. Paranoia
☐ No
☐ Yes

44. Confusion
☐ No
☐ Yes

45. Vivid Dreams
☐ No
☐ Yes

46. Hallucinations, drug-induced
☐ No
☐ Yes

47 Sleep Disorder
☐ No
☐ Yes

Exclusion Criteria

Criteria (check either 'Yes' as present or 'No' as absent)

48. Family History of a Similar Disorder
☐ No
☐ Yes

49. Secondary Cause by History
☐ No
☐ Yes

If Yes, Specify:

____

50. Secondary Cause by Investigation
☐ No
☐ Yes

If Yes, Specify:

____

51. Hallucinations, unrelated to Drugs
☐ No
☐ Yes

52. Dementia according to DSM IV
☐ No
☐ Yes

53. Slow Vertical Saccades
☐ No
☐ Yes

54. Vertical Gaze Palsy
☐ No
☐ Yes

55. Aphasia
☐ No
☐ Yes

56. Alien Limb Syndrome
☐ No
☐ Yes

Certain of MSA Diagnosis?
☐ No
☐ Yes

Sphincter EMG

58. Abnormal Anal Sphincter EMG
☐ Not performed
☐ Performed – Not abnormal
☐ Performed – Abnormal, please specify

If Abnormal, please specify:
____

59. Abnormal Urethral Sphincter EMG
☐ Not performed
☐ Performed – Not abnormal
☐ Performed – Abnormal, please specify

If Abnormal, please specify:
____

Imaging (check either “Yes” if the image was performed or “No” if it was not performed)

60. CCT
☐ No
☐ Yes

If Yes, Specify Results:
____

61. MRI
☐ No
☐ Yes

If Yes, Specify Results:
____

62. IBZM SPECT
☐ No
☐ Yes

If Yes, Specify Results:
____

63. Beta-CIT SPECT
☐ No
☐ Yes

If Yes, Specify Results:
____

64. FP-CIT SPECT
☐ No
☐ Yes

If Yes, Specify Results:
____

65. 18-F DOPA PET
☐ No
☐ Yes

If Yes, Specify Results:
____

66. FDG-PET
☐ No
☐ Yes

If Yes, Specify Results:
____

(check either 'Yes' or 'No')

67. Comorbidity
☐ No
☐ Yes

If Yes, Specify Results: ____________________________________

68.Antiparkinsonian Therapy
☐ No
☐ Yes

If Yes, Specify Results: ____________________________________

69. Concomitant Therapy
☐ No
☐ Yes

If Yes, Specify Results: ____________________________________
